# Genomic conservation and putative downstream functionality of the phosphatidylinositol signalling pathway in the cnidarian-dinoflagellate symbiosis

**DOI:** 10.3389/fmicb.2022.1094255

**Published:** 2023-01-26

**Authors:** Immy A. Ashley, Sheila A. Kitchen, Lucy M. Gorman, Arthur R. Grossman, Clinton A. Oakley, David J. Suggett, Virginia M. Weis, Sabrina L. Rosset, Simon K. Davy

**Affiliations:** ^1^School of Biological Sciences, Victoria University of Wellington, Wellington, New Zealand; ^2^Department of Marine Biology, Texas A&M University at Galveston, Galveston, TX, United States; ^3^Department of Plant Biology, The Carnegie Institution, Stanford, CA, United States; ^4^Climate Change Cluster, Faculty of Science, University of Technology Sydney, Broadway, NSW, Australia; ^5^Department of Integrative Biology, Oregon State University, Corvallis, OR, United States

**Keywords:** phosphatidylinositol, symbiosis, coral, inositol, Symbiodiniaceae, bioinformatics, reef, signalling

## Abstract

The mutualistic cnidarian–dinoflagellate symbiosis underpins the evolutionary success of stony corals and the persistence of coral reefs. However, a molecular understanding of the signalling events that lead to the successful establishment and maintenance of this symbiosis remains unresolved. For example, the phosphatidylinositol (PI) signalling pathway has been implicated during the establishment of multiple mutualistic and parasitic interactions across the kingdoms of life, yet its role within the cnidarian-dinoflagellate symbiosis remains unexplored. Here, we aimed to confirm the presence and assess the specific enzymatic composition of the PI signalling pathway across cnidaria and dinoflagellates by compiling 21 symbiotic anthozoan (corals and sea anemones) and 28 symbiotic dinoflagellate (Symbiodiniaceae) transcriptomic and genomic datasets and querying genes related to this pathway. Presence or absence of PI-kinase and PI-phosphatase orthologs were also compared between a broad sampling of taxonomically related symbiotic and non-symbiotic species. Across the symbiotic anthozoans analysed, there was a complete and highly conserved PI pathway, analogous to the pathway found in model eukaryotes. The Symbiodiniaceae pathway showed similarities to its sister taxon, the Apicomplexa, with the absence of PI 4-phosphatases. However, conversely to Apicomplexa, there was also an expansion of homologs present in the PI5-phosphatase and PI5-kinase groups, with unique Symbiodiniaceae proteins identified that are unknown from non-symbiotic unicellular organisms. Additionally, we aimed to unravel the putative functionalities of the PI signalling pathway in this symbiosis by analysing phosphoinositide (PIP)-binding proteins. Analysis of phosphoinositide (PIP)-binding proteins showed that, on average, 2.23 and 1.29% of the total assemblies of anthozoan and Symbiodiniaceae, respectively, have the potential to bind to PIPs. Enrichment of Gene Ontology (GO) terms associated with predicted PIP-binding proteins within each taxon revealed a broad range of functions, including compelling links to processes putatively involved in symbiosis regulation. This analysis establishes a baseline for current understanding of the PI pathway across anthozoans and Symbiodiniaceae, and thus a framework to target future research.

## 1. Introduction

Reef-building corals thrive in oligotrophic waters and form the basis of coral reef ecosystems. The success of this highly productive ecosystem relies on the symbiotic relationship formed between the cnidarian host (stony corals) and dinoflagellates of the family Symbiodiniaceae (LaJeunesse et al., 2004, 2018), whereby this symbiosis is underpinned by the mutual exchange of metabolic products. Host corals provide protection from the environment and supply the inorganic compounds needed for photosynthesis, whereas the symbionts provide the host with the majority of its metabolic carbon needs and enable efficient retention and recycling of nutrients (e.g., nitrogen) within the holobiont (Tanaka et al., 2006; Burriesci et al., 2012; Rädecker et al., 2015). Despite their biological and societal significance, coral reefs face unprecedented levels of degradation at a global scale (Frieler et al., 2013; Hughes et al., 2017). In particular, coral-dinoflagellate symbioses are sensitive to elevated temperatures, such that climate-driven ocean warming in the tropics is triggering increasingly frequent episodes of dysbiosis (termed “coral bleaching”) *en masse* (Frieler et al., 2013; Hughes et al., 2017).

Symbiodiniaceae taxa have evolved diverse physiological thresholds to environmental factors (Abrego et al., 2008; Howells et al., 2012; Hoadley et al., 2021; Nitschke et al., 2022). Certain Symbiodiniaceae species may confer thermal-tolerance to their host when harboured in abundance (Berkelmans and Van Oppen, 2006; Sampayo et al., 2008; Oliver and Palumbi, 2011; Claar et al., 2020), yet the molecular and physiological characteristics that underpin the specificity of symbioses between distinct Symbiodiniaceae taxa and host coral species remain poorly understood. Of particular interest are the molecular-level signalling events that lead to the successful establishment and maintenance of this symbiosis. Discovery-based analyses using diverse “omics” techniques have highlighted specific molecular pathways that likely play a significant role in regulating the symbiotic state (Lehnert et al., 2014; Rosic et al., 2015; Oakley et al., 2016; Bellantuono et al., 2018; Sproles et al., 2018a,b; Rosset et al., 2021), however, the exact functions of these pathways in the maintenance and establishment of the symbiosis are currently unknown.

**FIGURE 1:**
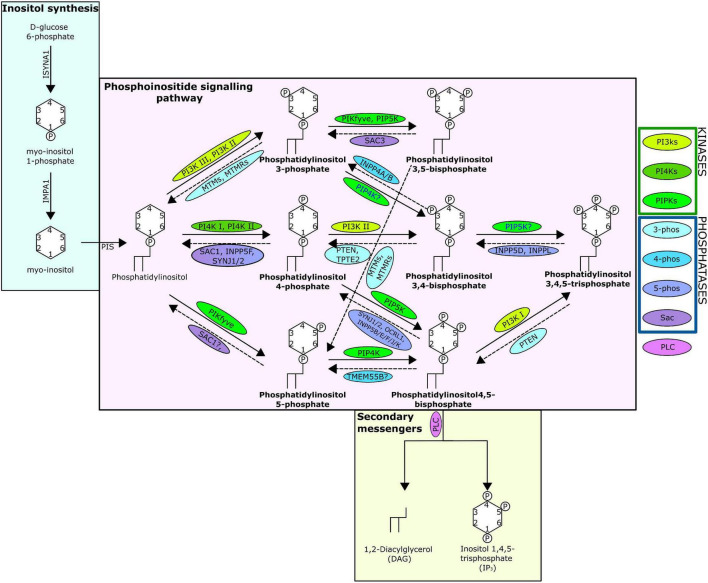
The phosphatidylinositol pathway as known from human studies. Solid arrows indicate kinase action; dashed indicate phosphatase action. Arrows are labelled with the kinases/phosphatases responsible for the reaction, question marks show where there are ambiguities surrounding the reaction, and the proteins are surrounded with a bubble coloured to indicate protein grouping from the key. The seven PIPs are indicated in bold and the structure of the lipids including the location and number of phosphates are shown. The absence of INPP5A is due to evidence suggesting no lipid phosphatase action (Pathway specifics derived from Dickson and Hille, 2019; Nakada-Tsukui et al., 2019).

One mechanism through which cellular messages are conveyed is by the phosphorylation and dephosphorylation of targeted compounds and proteins. This addition and removal of phosphate groups is carried out by the activities of kinase and phosphatase family enzymes, respectively. A key intracellular pathway that is activated through phosphorylation is the phosphatidylinositol (PI) signalling pathway (Figure 1). PI is among the least abundant of the mammalian phospholipids and is produced in the endoplasmic reticulum, before being transported to other cellular locations by phosphoinositide (PIP) transfer proteins (Dickson and Hille, 2019; Nakada-Tsukui et al., 2019). Coordinated activities of diverse PI kinases and phosphatases result in the production of seven distinct PIPs (Nakada-Tsukui et al., 2019) that occupy distinct locations within the cell, each enriched within a particular intracellular membrane (Dickson and Hille, 2019). These PIPs can act by binding directly to target proteins, referred to here as PIP-binding proteins. PIPs can also influence the membrane they embed in, or act as precursors to secondary messengers, known as inositol polyphosphates (IPPs) (Heilmann, 2016). PIPs have a wide variety of roles, regulating key cellular processes by triggering cellular signalling cascades, including those involved in immunity, apoptosis, vesicular trafficking, transmembrane signalling, ion channel regulation, lipid homoeostasis, and organelle identification (Underhill and Ozinsky, 2002; Di Paolo and De Camilli, 2006; Gericke et al., 2006; Thapa et al., 2012; Cernikova et al., 2020).

Several studies indicate that modification of PI signalling pathway activity plays roles in host-microbe interactions. Parasitic apicomplexans—the sister taxon to Symbiodiniaceae—modify their host’s cells to ensure their own survival, using molecular mechanisms such as the excretion of effector proteins into the host cell to sabotage host signalling and thereby prevent the host from mounting an immune response (Plattner and Soldati-Favre, 2008; Lüder et al., 2009). PI signalling is important in regulating the excretion of proteins that facilitate host cell invasion by *Plasmodium falciparum* (Ebrahimzadeh et al., 2019). Additionally, apicomplexans of the genus *Plasmodium* utilise the PI3K/Akt component of the PI pathway to successfully infect the host; inhibition of *Plasmodium* PI3K (phosphatidylinositol 3-kinase) in this instance, strongly decreases successful infection (Leirião et al., 2005). Also, through the pharmacological inhibition of phosphatidylinositol 4-kinase (PI4K) in *Plasmodium*, the infection process can be prevented at all stages of the parasitic lifecycle (McNamara et al., 2013). Modification of host cell PI3K activity by *Theileria* functions to ensure host cell proliferation and parasite survival (Baumgartner et al., 2000). Various bacterial pathogens also target the host PI signalling pathway through the activity of effector proteins to facilitate host cell entry and intracellular survival (Ham et al., 2011).

Multiple transcriptomic and proteomic studies have shown that several proteins involved in the PI signalling pathway are differentially expressed in both cnidarian hosts and Symbiodiniaceae in response to symbiosis (Lehnert et al., 2014; Rosic et al., 2015; Weston et al., 2015; Hillyer et al., 2016; Matthews et al., 2017; Bellantuono et al., 2018; Sproles et al., 2018a; Maor-Landaw et al., 2020). Collectively, this evidence indicates that the regulation of the PI signalling pathway in either or both host and symbiont may be important in the establishment and maintenance of the cnidarian-dinoflagellate symbiosis. However, the exact roles of the PI signalling pathway in the interactions between Symbiodiniaceae and cnidarians, and the effects on the maintenance and regulation of this symbiosis are unknown. Yet, a targeted study of such a specific molecular pathway in non-model organisms is confined by poor gene annotation and resulting lack of complete information on the genes involved.

Here we conduct a comprehensive bioinformatic analysis to identify proteins involved in the PI signalling pathway across multiple anthozoan and Symbiodiniaceae species, providing the first composite analysis of the PI signalling pathway within the cnidarian-dinoflagellate symbiosis. Additionally, we assess functionality of identified PIP-binding proteins to understand what downstream cellular pathways and processes are affected by the activity of the PI signalling pathway across these organisms, and hence potential mechanisms by which the PI pathway regulates the establishment and maintenance of this symbiosis.

## 2. Materials and methods

### 2.1. Dataset acquisition and quality assessment

**FIGURE 2:**
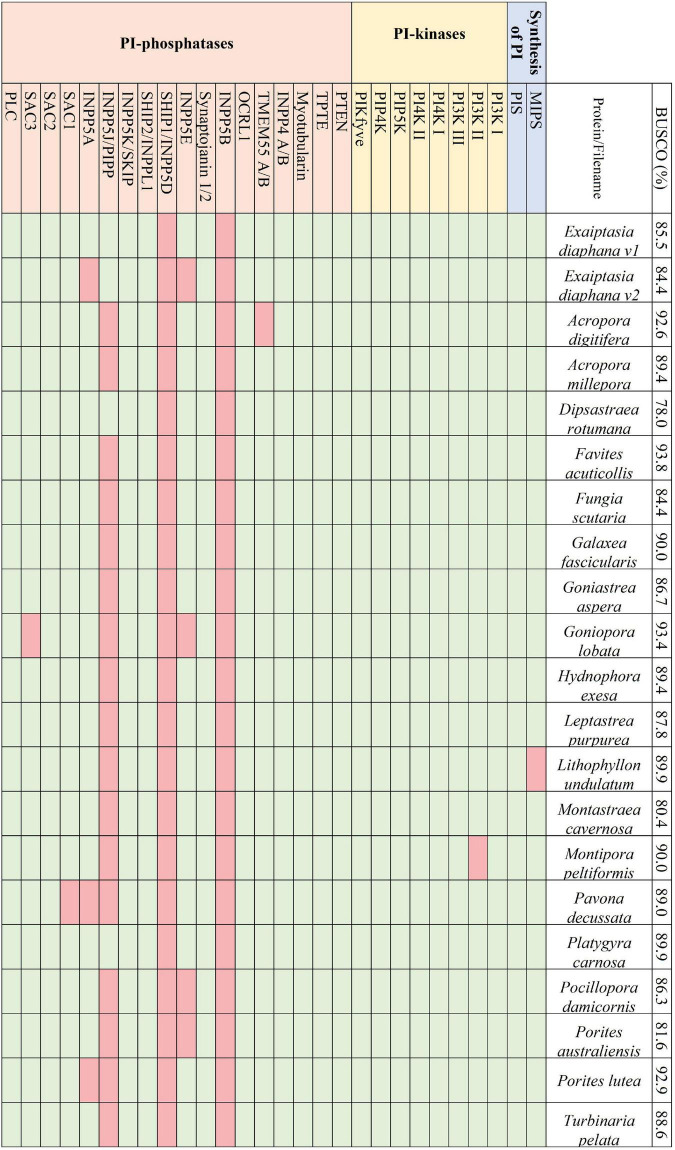
The presence and absence of the proteins constituting the PI pathway across the 21 selected symbiotic anthozoan assemblies and the respective BUSCO scores.

**FIGURE 3:**
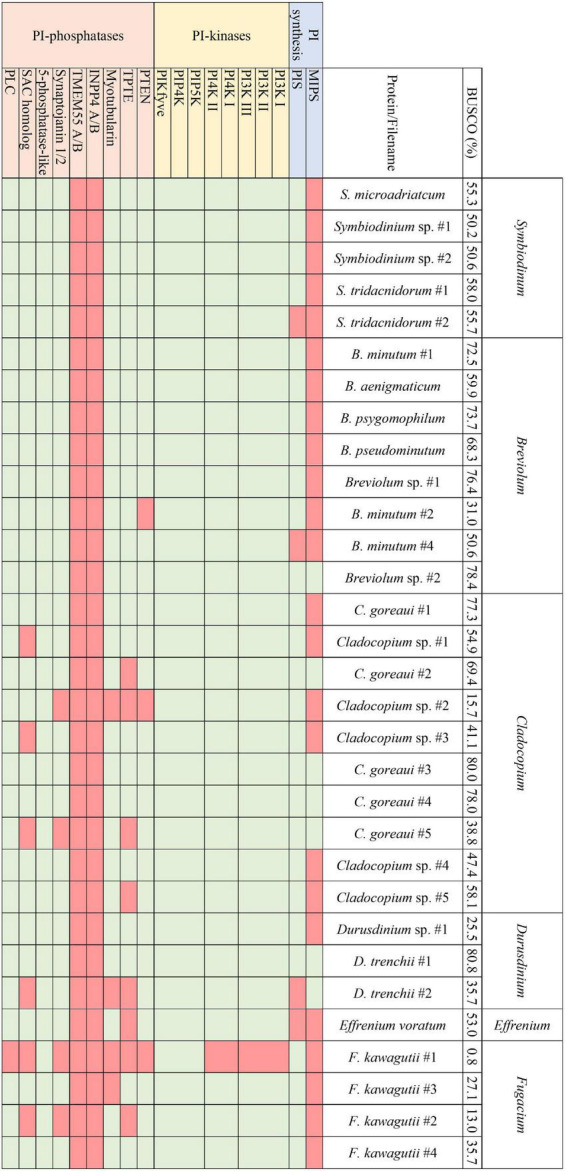
The presence and absence of the proteins constituting the PI pathway across all available Symbiodiniaceae assemblies and the respective BUSCO scores.

Transcriptomes and genomes of Symbiodiniaceae and symbiotic anthozoan species were obtained from publicly available sources (Symbiodiniaceae—Table 1, anthozoans—Table 2). For anthozoan species, we selected the highest-quality gene sets which also covered a range of hexacorals and *Exaiptasia diaphana* (*n* = 21 datasets). Due to the reduced number of available datasets for Symbiodiniaceae, all available assemblies (*n* = 28) were analysed, regardless of their quality. This spanned six genera, with most covering associations with hexacorals. BUSCO analysis, a metric to measure genome/transcriptome completeness through the identification of near-ubiquitous single-copy orthologs, was used to quantify the quality of these datasets (Manni et al., 2021). The range of BUSCO scores within the cnidarian datasets using the eukaryote database (v5) was between 78 and 93.8% complete, with an average of 87.8% (Figure 2). BUSCO completeness scores of the Symbiodiniaceae datasets using the eukaryote database (v5) ranged from 0.8 to 80.8%, with an average score of 49.2% (Figure 3). There was a particular focus throughout this analysis on the sea anemone *Exaiptasia diaphana* (“Aiptasia”), a widely used model organism for cnidarian-dinoflagellate symbiosis that can be maintained in an aposymbiotic state and re-colonised with multiple Symbiodiniaceae species (Weis et al., 2008).

**TABLE 1:** The Symbiodiniaceae datasets used for analysis.

Genus	Species	In-paper reference	Source	References
*Symbiodinium*	*Symbiodinium microadriaticum*	*Symbiodinium* sp. #1	Reefgenomics	Aranda et al., 2016
	*Symbiodinium* sp.	*Symbiodinium* sp. #2	iMicrobe	Keeling et al., 2014
	*Symbiodinium* sp. (CassKB8)	*Symbiodinium* sp. #3	Bayer et al., 2012	
	*Symbiodinium tridacnidorum* (CCMP2430)	*Symbiodinium* sp. #4	iMicrobe	Keeling et al., 2014
	*Symbiodinium tridacnidorum*	*Symbiodinium* sp. #5	Shoguchi et al., 2018	
*Breviolum*	*Breviolum minutum*	*Breviolum minutum* #1	Reefgenomics	Parkinson et al., 2016
	*Breviolum aenigmaticum*	*Breviolum aenigmaticum*	Reefgenomics	Parkinson et al., 2016
	*Breviolum psygomophilum*	*Breviolum psygomophilum*	Reefgenomics	Parkinson et al., 2016
	*Breviolum pseudominutum*	*Breviolum pseudominutum*	Reefgenomics	Parkinson et al., 2016
	*Breviolum* sp. (SSB01)	*Breviolum* sp. #1	Xiang et al., 2015	
	*Breviolum minutum*	*Breviolum minutum* #2	Bayer et al., 2012	
	*Breviolum minutum*	*Breviolum minutum* #3	Shoguchi et al., 2013	
	*Breviolum* sp. B1	*Breviolum* sp. #2	Camp et al., 2022	
*Cladocopium*	*Cladocopium goreaui* C1	*Cladocopium goreaui* #1	Camp et al., 2022	
	*Cladocopium* sp. C15	*Cladocopium* sp. #1	iMicrobe	Keeling et al., 2014
	*Cladocopium goreaui*	*Cladocopium goreaui* #2	iMicrobe	Keeling et al., 2014
	*Cladocopium* sp.	*Cladocopium* sp. #2	Ladner et al., 2012	
	*Cladocopium* sp.	*Cladocopium* sp. #3	González-Pech et al., 2017	
	*Cladocopium goreaui*—MI population	*Cladocopium goreaui* #3	Levin et al., 2016	
	*Cladocopium goreaui*—SM population	*Cladocopium goreaui* #4	Levin et al., 2016	
	*Cladocopium goreaui*	*Cladocopium goreaui* #5	Reefgenomics	Liu et al., 2018
	*Cladocopium* sp. C92	*Cladocopium* sp. #4	(Shoguchi et al., 2018)	
	*Cladocopium* sp.	*Cladocopium* sp. #5	iMicrobe	Keeling et al., 2014
*Durusdinium*	*Durusdinium* sp.	*Durusdinium* sp. #1	Ladner et al., 2012	
	*Durusdinium trenchii* D1a	*Durusdinium trenchii* #1	Camp et al., 2022	
	*Durusdinium trenchii*	*Durusdinium trenchii* #2	iMicrobe	Keeling et al., 2014
*Effrenium*	*Effrenium voratum* (CCMP421)	*Effrenium voratum*	iMicrobe	Keeling et al., 2014
*Fugacium*	*Fugacium kawagutii* (CCMP2468)	*Fugacium kawagutii* #1	iMicrobe	Keeling et al., 2014
	*Fugacium kawagutii* v2	*Fugacium kawagutii* #3	SAGER	Liu et al., 2018
	*Fugacium kawagutii* v1	*Fugacium kawagutii* #2	SAGER	Lin et al., 2015
	*Fugacium kawagutii* v3	*Fugacium kawagutii* #4	SAGER	Li et al., 2020

**TABLE 2:** The anthozoan datasets used for analysis.

Species	Source	References
*Exaiptasia diaphana v1*	Reefgenomics	Baumgarten et al., 2015
*Exaiptasia diaphana v2*	Reefgenomics	Baumgarten et al., 2015
*Acropora digitifera*	CoralTBase	Zhang et al., 2019
*Acropora millepora*	Reefgenomics	Bhattacharya et al., 2016
*Dipsastraea rotumana*	CoralTBase	Zhang et al., 2019
*Favites acuticollis*	CoralTBase	Zhang et al., 2019
*Fungia scutaria*	Reefgenomics	Bhattacharya et al., 2016
*Galaxea fascicularis*	CoralTBase	Zhang et al., 2019
*Goniastrea aspera*	Reefgenomics	Voolstra et al., 2015
*Goniopora lobata*	CoralTBase	Zhang et al., 2019
*Hydnophora exesa*	CoralTBase	Zhang et al., 2019
*Leptastrea purpurea*	CoralTBase	Zhang et al., 2019
*Lithophyllon undulatum*	CoralTBase	Zhang et al., 2019
*Montastraea cavernosa*	Reefgenomics	Bhattacharya et al., 2016
*Montipora peltiformis*	CoralTBase	Zhang et al., 2019
*Pavona decussata*	CoralTBase	Zhang et al., 2019
*Platygyra carnosa*	CoralTBase	Zhang et al., 2019
*Pocillopora damicornis*	Reefgenomics	Cunning et al., 2018
*Porites australiensis*	Reefgenomics	Bhattacharya et al., 2016
*Porites lutea*	CoralTBase	Zhang et al., 2019
*Turbinaria pelata*	CoralTBase	Zhang et al., 2019

### 2.2. Bioinformatic identification of phosphatidylinositol signalling pathway proteins

A literature search was used to identify conserved domains of the PI kinases and phosphatases. The conserved domains were used to search the genomic and transcriptomic resources using the “aphid” R-package (Wilkinson, 2018). Specifically, 17 Hidden Markov Models (HMMs) that provided complete coverage of the PI signalling pathway were used as queries to identify proteins of interest (Table 3). Resulting proteins with a score >20 (an observed cut-off in quality and accuracy below this score) were run through the InterProScan plugin in Geneious (v2019.2.3) to identify additional domains. To verify the presence or absence of PI pathway genes, we also used: (1) a custom BLAST analysis against a database of the 28 Symbiodiniaceae and 21 cnidarian species (*E*-value cut-off 10^–2^); (2) motif identification through thorough literature searches; (3) protein alignments using Muscle in Geneious; and (4) similarity scores produced during alignments. Moreover, genes identified above were assigned to gene families, or orthogroups, using OrthoFinder 2.5.2 (Emms and Kelly, 2015). In complex cases, such as in the distinction between phosphatidylinositol 3- and 4-kinases, where the same domain is used to extract distinct target proteins of interest, maximum-likelihood phylogenetic trees were reconstructed using the conserved protein domains with RAxML (v4.0 in Geneious, Protein model: Gamma Blosum 62) (Supplementary Figure 1).

**TABLE 3:** The domains targeted to provide complete coverage of the PI pathway, the targeted proteins of these domains, and the Pfam identifiers.

Domain targets	Protein targets	Pfam identifiers
inos-1-Psynth	Inositol-3-phosphate synthase	PF01658
CDP	PI synthase	PF01066
PI3_PI4_kinase	PI 3- and 4-kinases	PF00454
PIPK	PI 5-kinases	PF01504
PTEN_C_2_	TPTE and PTEN	PF10409
myotub_related	Myotubularins	PF06602
C_2_	INPP4 A/B	PF00168
Tmemb_55A	TMEM55 A/B	PF09788
Syja_N	Synaptojanin 1/2	PF02383
ASH	OCRL	PF15780
Exo_endo_phos	Synaptojanin 1/2, INPP5 (A, B, D, E, L1), OCRL, SKIP 1/2	PF03372
hSac2	SAC 1/2/3	PF12456
OCRL_clath_bd	OCRL	PF16726
RhoGAP	INPP5B, OCRL	PF00620
SH2	INPP5L1, INPP5D	PF00017
PI-PLC-X, PI-PLC-Y	PI-specific phospholipase C	PF00387, PF00388

Blue indicates precursor proteins to the PI signalling pathway, yellow the PI-kinases, orange the PI-phosphatases and purple PLC.

### 2.3. OrthoFinder analysis

OrthoFinder was used for ortholog assignment of cnidarians and Symbiodiniaceae separately (Emms and Kelly, 2015). Four different analyses were carried out: comparison within the available Symbiodiniaceae datasets; within selected symbiotic cnidarians; within symbiotic and non-symbiotic cnidarians; and within the Symbiodiniaceae plus non-symbiotic unicellular organisms (for symbiotic/non-symbiotic comparison). Protein groups were further delineated by similarity to each other using BLAST, which was especially useful when analysing Symbiodiniaceae datasets, and when attempting to identify differences in the identifiable pathways between symbiotic and non-symbiotic cnidarians/Symbiodiniaceae.

### 2.4. Bioinformatic analysis of phosphoinositide (PIP)-binding protein functionality

The methods described above were used to identify proteins involved in PIP-binding within datasets of the target species *E. diaphana* and *Breviolum minutum*, as well as in datasets of selected species spanning a variety of symbiotic/non-symbiotic symbiont and cnidarian types (Table 4). *E. diaphana* and *B. minutum* were utilised due to their common association in nature and the use of them as a model organism (Weis et al., 2008). Previous literature on PIP-binding proteins were used to identify regions of conservation (HMMs) (Cernikova et al., 2019, 2020). Within each of these databases, 14 HMMs were queried. Proteins with at least one of the required PIP-binding domains were included in the gene enrichment analysis (Table 5). The PIP-binding proteins for each species were run through Pannzer2 to ascertain Gene Ontology (GO) terms (Törönen et al., 2018). The “TopGO” R-package (Alexa and Rahnenfuhrer, 2016) was used for the gene enrichment analysis to compare the identified PIP-binding proteins to the entire GO repertoire of biological processes, molecular functions, and cellular compartments within each target species. The GO terms with adjusted *p*-value < 0.05 were further condensed using Revigo semantic similarity clustering (Supek et al., 2011) (Settings: Small (0.5) similarity, *p*-value, whole Uniprot database, “SimRel”: a semantic similarity measure). A literature search was then completed to find a link for the significant biological terms to either a mutualistic or parasitic interaction.

**TABLE 4:** The non-symbiotic species datasets used for analysis.

Species	Source	References
*Actinia equina*	Reefgenomics	Wilding et al., 2020
*Alexandrium tamarense*	iMicrobe	Moustafa et al., 2010
*Amphidinium massartii*	iMicrobe	Murray et al., 2004
*Amphimedon queenslandica*	Reefgenomics	Bhattacharya et al., 2016
*Chlorella sorokiniana*	Arriola et al., 2018	
*Ephydatia muelleri*	Reefgenomics	Bhattacharya et al., 2016
*Nematostella vectensis*	Reefgenomics	Bhattacharya et al., 2016
*Plasmodium falciparum*	UniProt	Bateman et al., 2021
*Scrippsiella hangoei*	iMicrobe	Keeling et al., 2014
*Tubastraea coccinea*	CoralTBase	Zhang et al., 2019

**TABLE 5:** The domains targeted to provide complete coverage of the proteins that act upon the PI pathway (PIP-binding proteins).

Domain targets	Pfam identifiers
ANTH	PF07651
BAR	PF03114
C_2_	PF00168
ENTH	PF01417
FERM	PF00373
FYVE	PF01363
GOLPH3/GPP34	PF05719
NECAP/DUF1681	PF07933
PDZ	PF00595
PH	PF00169
PROPPIN/WD40 repeat	PF00400
PTB	PF08146
PX	PF00787
Tub	PF01167

## 3. Results and discussion

### 3.1. The phosphatidylinositol pathway in anthozoan datasets

Across the symbiotic anthozoans analysed there was a complete and conserved PI pathway, analogous to that found in multicellular eukaryotic model organisms (Michell, 2011; Balla, 2013; Figure 2). PI3- and PI4- kinases constitute enzymatic families in higher eukaryotes that are subdivided into multiple classes. Members from each group of the kinases as well as members of the 3- and 4-phosphatases were also identified across the surveyed anthozoans. Phylogenetic trees constructed for the PI3- and PI4-kinases identified each kinase subclass, suggesting the conservation of these kinases within Anthozoa (Supplementary Figure 1). Conversely, 5-phosphatases were not conserved throughout the symbiotic anthozoans, with the loss of INPP5B and SHIP1 in all taxa, while others such as SKIP, PIPP, INPP5E and inositol 1,4,5-trisphosphate 5-phosphatase (INPP5A) were absent from various lineages (Figure 2).

We did not identify any direct evidence of altered regulation of anthozoan host PI-kinases and -phosphatases in response to symbiosis in published datasets. Yet, the mechanism of activation of these enzymes—by phosphorylation—means that more commonly used “omics” technologies (transcriptomics, proteomics) may not identify altered activity in the PI signalling pathway. Interestingly, however, sodium and proton myo-inositol co-transporters are upregulated in symbiotic Aiptasia at the transcriptome (Lehnert et al., 2014) and proteome levels (Sproles et al., 2018b). Myo-inositol is the biologically active precursor to the PI pathway that plays a central role in signal transmission (Figure 1). Metabolomic analysis also found the accumulation of inositol in thermally stressed anemones, indicating that its clearance is important for maintaining the cnidarian-dinoflagellate symbiosis (Hillyer et al., 2016). Furthermore, proteomic analysis identified down-regulation of multiple sugar-inositol transporters, the proton myo-inositol co-transporter (HMIT), the myo-inositol/Na + co-transporter (SMIT1), and the myo-/chiro-inositol/glucose transporter (SMIT2) when the host was inoculated with the heat tolerant, but physiologically sub-optimal (Sproles et al., 2018b) symbiont *Durusdinium trenchii* (Matthews et al., 2017). Taken together, this suggests that the efficient transport of myo-inositol is essential for the establishment and maintenance of an optimal symbiosis.

### 3.2. The phosphatidylinositol pathway in Symbiodiniaceae datasets

Analogous to the PI pathway found within Apicomplexa (Wengelnik et al., 2018; Cernikova et al., 2019; Nakada-Tsukui et al., 2019; Cestari and Stuart, 2020), Symbiodiniaceae displayed no genes of the 4-phosphatase group (Figure 3). However, conversely to apicomplexans, Symbiodiniaceae showed a greatly increased diversity both of and within the 5-phosphatases, with multiple 5-phosphatase homologs with dual domain structure, identified within the majority of Symbiodiniaceae species (Supplementary Tables 1, 2). Anthozoan genomic and transcriptomic data showed an average of four and three proteins with the PIPK (used for identification of PIP5K proteins) domain, respectively. However, Symbiodiniaceae genomic and transcriptomic datasets both showed an average of 15 proteins containing the PIPK domain, despite the reduced BUSCO scores. As the lowest BUSCO score for anthozoans was 78%, we used that as a threshold to look at comparative numbers of Symbiodiniaceae PIPK domain-containing proteins, and the average proteins further increased to 21. Of particular note was the presence of a dual domain protein within 15 of the Symbiodiniaceae datasets, with both the PIPK domain and also the PI-PLC domain. Searches of the BLAST database showed no results for similar proteins in other species. The range of PI kinases discovered was diverse, with members from each kinase group identified in all but one Symbiodiniaceae species (Figure 3). The exception was “*F. kawagutii* #1” —a dataset of *Fugacium kawagutii*—that only returned a 0.8% complete BUSCO score. However, the lack of functional annotation of proteins from other dinoflagellates makes identification difficult. We relied primarily on sequence homology to the characterised 5-phosphatases (*n* = 10) and other well-annotated results achieved through NCBI BLAST analysis. Caution should be exercised when examining gene absence in this group because of the incompleteness of some of the datasets used (BUSCO scores found in Figure 3). Despite the sparseness of the available datasets, we were able to identify a completely functional PI pathway across the analysed Symbiodiniaceae species.

Transcriptomic analysis of four species spanning a diversity of four Symbiodiniaceae genera [*Symbiodinium* (ITS strain A2), *Breviolum* (ITS strain B2), *Cladocopium* (ITS strain C1), and *Durusdinium* (ITS strain D1)] revealed that six cellular pathways are common to all four genera, including the PI signalling pathway and the inositol phosphate metabolism pathway, highlighting the potential global significance of these pathways in Symbiodiniaceae (Rosic et al., 2015). Notable genes encoding phosphatidylinositol 4-phosphate 5-kinases (PIP5K) and phosphatidylinositol 4-kinase (PI4K) were identified across all four Symbiodiniaceae species analysed. PIP4K synthesises PI 4-phosphate (PI4P), which is vital for the regulation of chloroplast division (Bovet et al., 2001; Okazaki et al., 2015). The ubiquity of PIP4K across the Symbiodiniaceae datasets analysed here is therefore unsurprising, with exceptions likely reflecting the quality of available resources. Meanwhile, PIP5K is the main route for the production of phosphatidylinositol 4,5-bisphosphate, and has cellular roles in actin dynamics, endocytosis, exocytosis, and focal adhesion assembly (Tan et al., 2015; Nakada-Tsukui et al., 2019). PIP5K also plays an important role in endosomal trafficking, with the specific isoform PIPKIyi2 being localised to recycling endosomes to facilitate transmembrane protein recycling (Thapa et al., 2012). Exocytosis and endocytosis are important functions in signalling processes within symbiosis (Weis, 2008; Oakley et al., 2016; Rosset et al., 2021) and hence, the down-regulation of this protein when the alga is in culture could reflect an increased demand for vesicle transport after colonisation of a host. Meanwhile PIP4K synthesises PI 4-phosphate (PI4P), which is vital for the regulation of chloroplast division (Bovet et al., 2001; Okazaki et al., 2015). The ubiquity of PIP4K and PIP5K across the Symbiodiniaceae datasets analysed here is therefore unsurprising, with exceptions likely reflecting the quality of available resources.

A transcriptomic study of *B. minutum* demonstrated a significant down-regulation of PIP5K activity when the alga was in culture compared to *in hospite*, providing further support for the importance of PIP5K in the functionality of symbiosis (Maor-Landaw et al., 2020). A similar transcriptomic analysis explored the differences between the transcriptome of the thermally tolerant *D. trenchii* when free-living and *in hospite* in Aiptasia (Bellantuono et al., 2018). Our targeted analysis of the dataset from Bellantuono et al. (2018) revealed that many of the PI pathway-related genes were significantly differentially expressed between the symbiont when *in hospite* and in culture (Supplementary Figures 2A, B). Notably there was a large downregulation of PIP5K “2” and a large upregulation of PIP5K “9” and “7,” among other types, when the symbiont was in symbiosis compared to free-living. These observations of up-regulated PIP5K expression in symbiosis could reflect an increased demand for vesicle transport in symbiosis. It is also possible that, as observed in other host-microbe interactions (Baumgartner et al., 2000; Drecktrah et al., 2004; Leirião et al., 2005; Ham et al., 2011; Ebrahimzadeh et al., 2019), Symbiodiniaceae modifies PI-kinase expression as a molecular strategy to manipulate host PIP synthesis to facilitate host membrane dynamics and regulate cellular mechanisms essential for symbiosis persistence.

### 3.3. Ortholog differences between non-symbiotic and symbiotic groups

The range of proteins within the PI pathway was highly conserved between symbiotic and non-symbiotic cnidarians (Tables 1, 2 and Supplementary Table 3). Both coverage of the pathway and number of proteins within each ortholog were similar, with no significant increases or decreases in gene numbers between cnidarians that associate with symbionts (Supplementary Tables 3, 4).

In contrast, differences were observed between the Symbiodiniaceae and non-symbiotic unicellular algal datasets, including presence/absence of ortholog groups and varying numbers of isoforms/duplicates of individual orthogroups between symbiotic and non-symbiotic datasets. One orthogroup of interest was OG0006447, which was absent in all five of the non-symbiotic species, but present across all except one of the Symbiodiniaceae datasets (“*F. kawagutii* #1” —previously noted to be largely incomplete based on BUSCO analysis). This orthogroup was analysed further using NCBI BLAST, where it matched consistently and with a high sequence conservation to a phosphatidylinositol 5-phosphate 4-kinase, or PIP5K/PIPK type II protein. This protein has been described across model metazoans but has not yet been described within unicellular organisms; indeed some research has suggested that it is not present in unicellular organisms (Mathre et al., 2019; Raghu, 2021). This protein is responsible for a small proportion of the production of PI 4,5-bisphosphate, however, it is predicted that its primary role is the regulation of PI 5-phosphate (PI5P), which acts as its binding substrate (Rameh et al., 1997; Raghu, 2021). This protein is localised to the plasma membrane, vesicular compartments and nucleus, with roles in PI3K-based regulation of receptor-activated signalling, membrane transport, autophagosome regulation, and the control of PI5P levels in response to cues such as UV irradiation and oxidative stress (Keune et al., 2013; Vicinanza et al., 2015; Wang et al., 2019; Raghu, 2021). Interestingly, the loss of this protein in mouse models leads to the hyperactivation of the innate immune system (Shim et al., 2016). The presence of this protein across all but one of the 28 Symbiodiniaceae datasets in our study is noteworthy, and experimental research to elucidate its role in symbiosis would be valuable.

Another orthogroup (OG0015044) predicted to be a PTEN/TPTE2 3-phosphatase homolog showed a similar pattern. Although there were other orthogroups that showed the presence of PTEN/TPTE2 ubiquitously across symbiotic and non-symbiotic unicellular organisms, this separate orthogroup (OG0015044) indicates an expansion of this protein type within the Symbiodiniaceae datasets. Most of the proteins within this orthogroup showed predominantly PTEN hits when run against the NCBI BLAST database. PTEN is a negative regulator of PI3K and has a variety of roles within cells, including survival, proliferation and regulation of cellular architecture, and it is frequently disrupted in cancer (Song et al., 2012). The presence of an expanded PTEN homolog group is observed in the parasitic protist, *Entamoeba histolytica*, which has six PTEN homologs, while there is only one present in humans (Nakada-Tsukui et al., 2019). The expansion of the PTEN group within Symbiodiniaceae is indicative of an importance of PTEN functionality, given its role in cell survival and proliferation, and it is therefore another good candidate for future functional studies of the cnidarian-dinoflagellate symbiosis.

A few notable Symbiodiniaceae orthogroups (e.g., OG0012793, OG0014270) were also distinct between Symbiodiniaceae and non-symbiotic unicellular organisms; however, the proteins did not BLAST to any known annotations, or recovered only a few hits, with “unnamed” or “hypothetical protein” responses. When analysed using InterPro scan, these orthogroups contained just one domain, PIPK. However, without targeted experimental work, these protein groups cannot be identified beyond the PIPK group in general.

### 3.4. Identification and predicted function of putative phosphoinositide-binding proteins in the Aiptasia-*Breviolum* symbiosis

To further understand the downstream functions of the PI pathway and to unravel the potential role in symbiosis, we expanded the analysis to include enrichment of proteins with the capabilities of binding to members of the PI pathway (termed PIP-binding proteins). All 14 identified domains of interest were found in Aiptasia, whereas four domains were absent in *Breviolum* (ANTH, FERM, GOLPH3, PTB). Within Aiptasia, 913 PIP-binding proteins were identified, which was 3.12% of the total predicted proteins in the genome, resulting in 306 significantly (*p* < 0.05) enriched GO terms (Supplementary Table 5). Within Symbiodiniaceae (*Breviolum* sp. B1), 898 PIP-binding proteins were identified, which was 0.91% of the total predicted proteins in the assembly, resulting in 103 significant GO terms (Supplementary Table 6).

**FIGURE 4:**
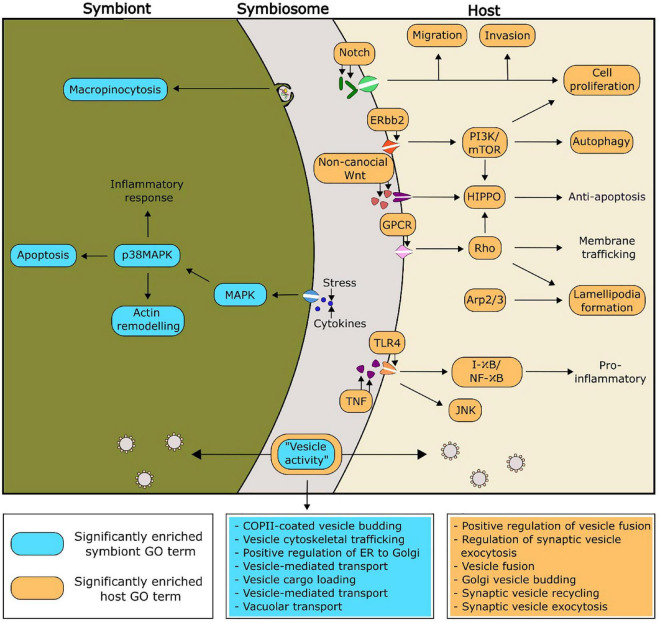
Proposed functionality within the symbiosis of selected significantly enriched functions of the PIP-binding proteins, based on enriched GO functions within the model cnidarian, Aiptasia (orange), and *Breviolum minutum* (blue). The GO terms related to vesicle activity have been expanded in the two boxes coloured blue and orange, for symbiont and host, respectively.

Putative functionalities of the PIP-binding proteins identified in Aiptasia and Breviolum that were predicted to be relevant to symbiosis regulation can be found in Figure 4. Of particular interest, there were seven and six significantly enriched GO terms related to vesicle functioning in *Breviolum* and Aiptasia, respectively. We hypothesise that cellular processes involving vesicles, such as vesicle-mediated transport, play an important role in the functioning of the cnidarian-dinoflagellate symbiosis.

### 3.5. Analysis of PIP-binding functionality in additional species of symbiotic anthozoans and Symbiodiniaceae

Enriched GO terms can be found in Supplementary Tables 7–20. The GO term “COPII-coated vesicle budding” was significantly enriched within one of the symbiotic cnidarians (the coral *Montipora peltiformis*), but more noticeably, all analysed Symbiodiniaceae. One role of COPII vesicles is the transport of proteins from the endoplasmic reticulum, the site of PI synthesis (Agranoff et al., 1958; Sato and Nakano, 2007; Balla, 2013), suggesting an important role of PI in the symbiont. Two GO terms corresponding to immune responses (“innate immune response” and “immune response-activating cell surface receptor”) were found to be significantly enriched across four out of the five Symbiodiniaceae species analysed, whereas these GO terms were not found to be enriched across any of the cnidarians analysed. There is evidence of endosymbiotic *Leishmania* spp. modulating their host’s immune system through the secretion of exosomes into the host (Silverman and Reiner, 2011). Other research shows *Leishmania* spp. subverting host immune responses by targeted host pathways such as the PI 3-kinase/Akt signalling cascade to prevent the activation of macrophages (Ruhland et al., 2007; Lambertz et al., 2012). This evidence extends to the cnidarian-dinoflagellate symbiosis, with 16% of differentially expressed genes between symbiotic states being composed predominantly of genes involved in apoptosis and oxidative stress; which are critical functions of immune responses (Kvennefors et al., 2010; Matthews et al., 2017).

Another GO term that regularly appeared as significant is the “positive regulation of TORC1 signalling” in four out of the five analysed Symbiodiniaceae, and “positive regulation of TOR signalling” in three out the five symbiotic cnidarians analysed. TOR (target of rapamycin) signalling activity inhibits autophagy (Juhász et al., 2008), and the presence of significantly enriched GO terms in both the symbiotic cnidarian group and Symbiodiniaceae indicates active inhibition of autophagy across both groups. Transcriptomics supports this finding, with elevated levels of mTORC1 kinase in symbiotic Aiptasia compared to non-symbiotic animals, suggesting a mechanism similar to a feeding response in symbiotic cnidarians (Voss et al., 2019). Up-regulation of TOR-related genes and transcripts during colonisation of plants by mycorrhizae suggests its role in other symbioses (Nanjareddy et al., 2016; Arthikala et al., 2021).

Understanding the implications for the enriched GO terms in the cnidarian-dinoflagellate symbiosis is complex, as just because a function does not appear with a high frequency does not mean its role in the symbiosis is unimportant. Further experimental work, including the inhibition and knock-out of specific genes, will help to elucidate the roles of these highlighted GO terms in the functioning and maintenance of the symbiosis.

## 4. Conclusion

Our bioinformatic analysis of PI-kinases, PI-phosphatases and PIP-binding proteins is the first to characterise the PI signalling pathway across anthozoans and Symbiodiniaceae. We thereby provide a platform to guide further—more targeted—research. For example, studies will need to apply high-throughput methodologies such as phosphoproteomics and targeted lipidomics to analyse PI pathway activity in cnidarians and Symbiodiniaceae, to ascertain the differences across different experimental treatments, during symbiosis establishment, and across a variety of different cnidarian-dinoflagellate pairings. Such approaches will enable us to begin to unlock the role of the PI signalling pathway in this ecologically important symbiosis. Elucidating how specific molecular processes such as PI signalling regulate the coral-dinoflagellate symbiosis is of high value to identify potential targets to engineer more resilient symbiotic associations [reviewed by Rosset et al., 2021)], a tool that may become necessary in the conservation and restoration of coral reefs.

## Data availability statement

The datasets presented in this study can be found in online repositories. The locations and references for the datasets used in this paper are available in Tables 1, 2, 4.

## Author contributions

IA, SD, AG, DS, VW, CO, and SR conceptualized the project. IA performed the analysis and wrote the manuscript. SK and LG assisted in data analysis. SD, AG, DS, VW, SR, CO, and SK contributed to the revisions. All authors read and approved the final manuscript.
