# Supplementary figures and images for: Genomic conservation and putative downstream functionality of the phosphatidylinositol signalling pathway in the cnidarian-dinoflagellate symbiosis
Source: Front Microbiol. 2023 Jan 26;13:1094255. doi: 10.3389/fmicb.2022.1094255 (PMC9909359; doi:10.3389/fmicb.2022.1094255)

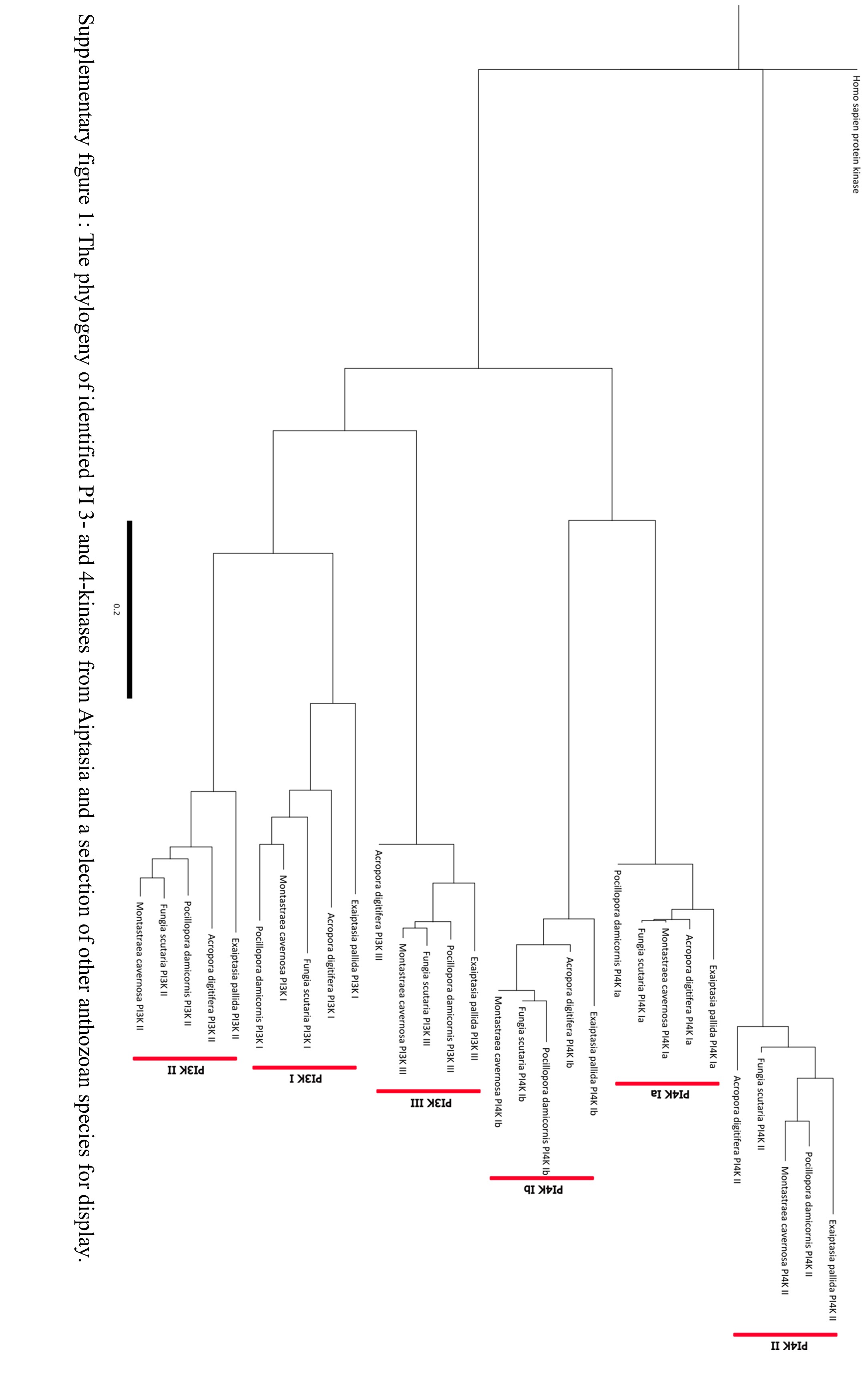

Supplement: Supplementary file 3 [file Image_1.jpg]

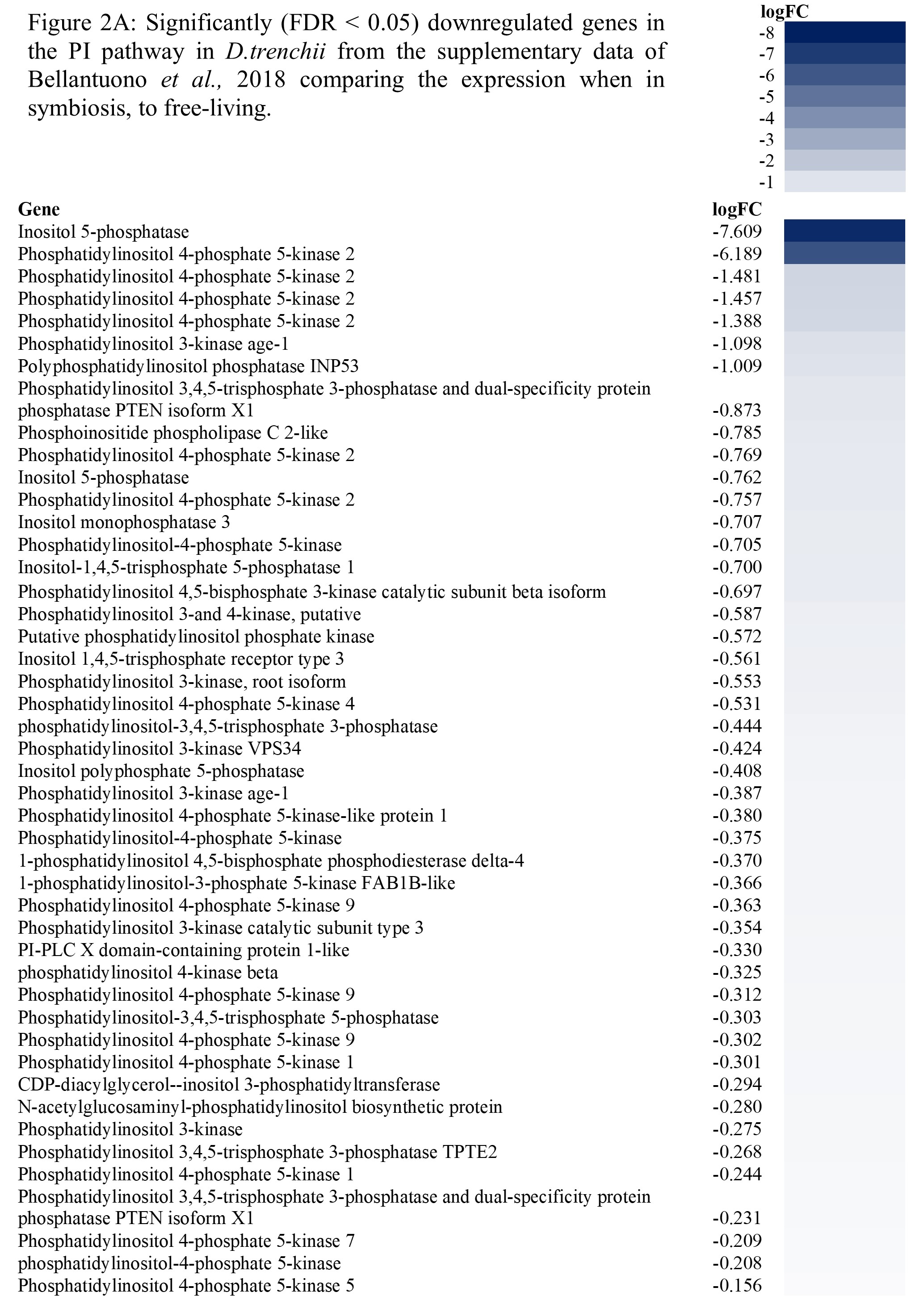

Supplement: Supplementary file 4 [file Image_2.jpg]

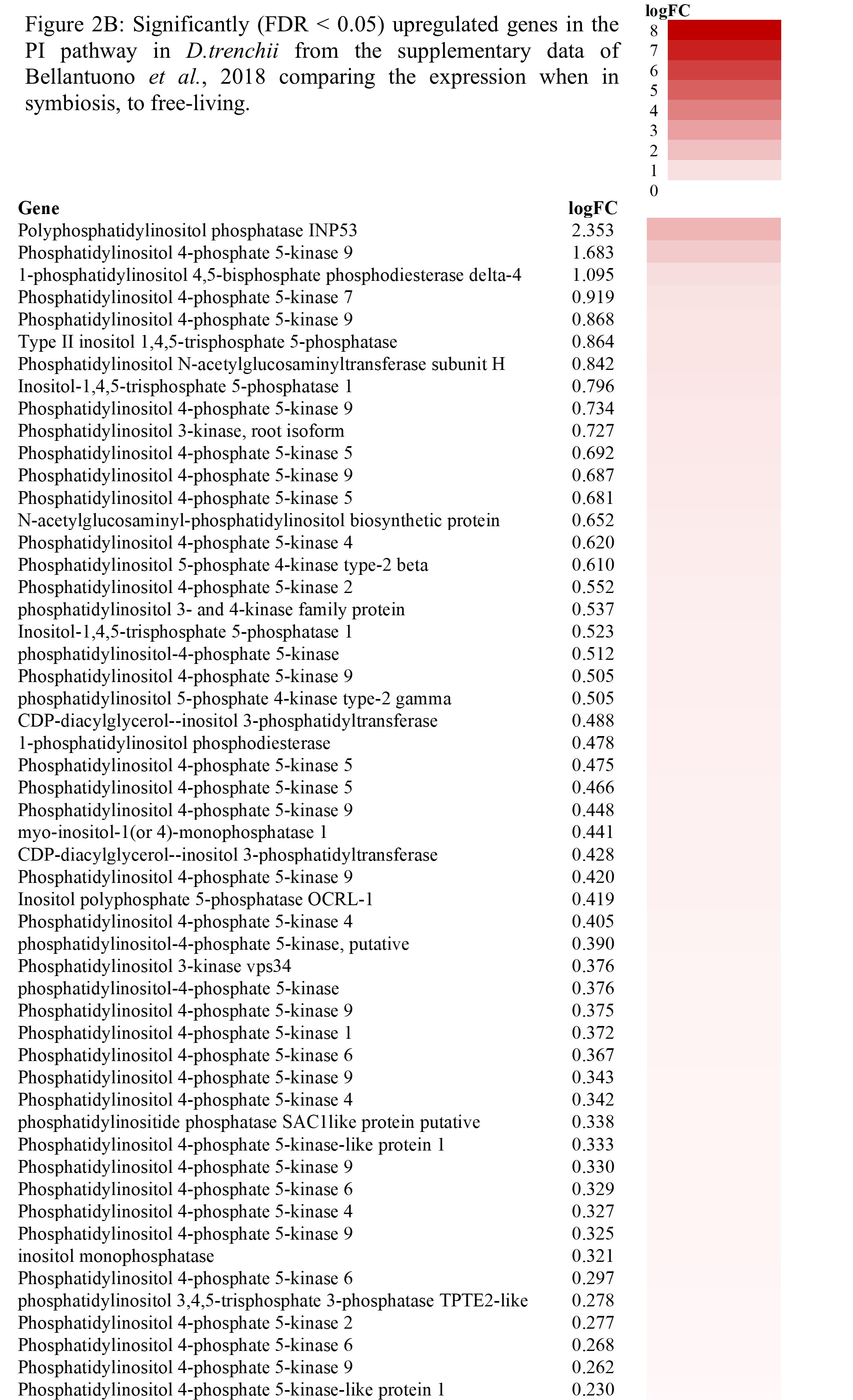

Supplement: Supplementary file 5 [file Image_3.jpg]
